# Supplementary material for: Population Heterogeneity in the Epithelial to Mesenchymal Transition Is Controlled by NFAT and Phosphorylated Sp1
Source: PLoS Comput Biol. 2016 Dec 27;12(12):e1005251. doi: 10.1371/journal.pcbi.1005251 (PMC5189931; doi:10.1371/journal.pcbi.1005251)
Supplement: S1 Text — (PDF) [file pcbi.1005251.s001.pdf]

Supplemental Materials and Methods

Characteristic transcription and translation parameters.

We used literature based transcription and translation parameters to establish the characteristic synthesis and degradation rates for both mRNA and protein. We estimated values for the rate parameters from the Bionumbers database [31]. These parameters were then used for all gene expression calculations:

```
------------------------------------------------------------------
# Description
------------------------------------------------------------------
cell_diameter = 12                        # mu m
number_of_rnapII = 75000                  # copies/cells
number_of_ribosome = 1e6                  # copies/cells
mRNA_half_life_TF = 2                     # hrs
protein_half_life = 10                    # hrs
doubling_time   = 19.5                    # hrs
max_translation_rate = 5                  # aa/sec
max_transcription_rate = 6.0              # nt/sec
average_transcript_length = 15000         # nt
average_protein_length = 5000             # aa
fraction_nucleus = 0.49                   # dimensionless
av_number = 6.02e23                       # number/mol
avg_gene_number = 2                       # number of copies of a gene
------------------------------------------------------------------
```

```
--------------------------------------------------------------------------------------
# Description
--------------------------------------------------------------------------------------
# Calculate the volume (units: L)
V = ((1-fraction_nucleus)*(1/6)*(3.14159)*(hl60_diameter)^3)*(1e-15)

# Calculate the rnapII_concentration and ribosome_concentration (units: nM)
rnapII_concentration = number_of_rnapII*(1/av_number)*(1/V)*1e9
ribosome_concentration = number_of_ribosome*(1/av_number)*(1/V)*1e9

# degradation rate constants (units: hr^-1)
degradation_constant_mRNA = -(1/mRNA_half_life_TF)*log(0.5)
degradation_constant_protein = -(1/protein_half_life)*log(0.5)

# kcats for transcription and translation (units: hr^-1)
kcat_transcription = max_transcription_rate*(3600/average_transcript_length)
kcat_translation = max_translation_rate*(3600/average_protein_length)

# Maximum specific growth rate (units: hr^-1)
maximum_specific_growth_rate = (1/doubling_time)*log(2)

# What is the average gene concentration (units: nM)
avg_gene_concentration = avg_gene_number*(1/av_number)*(1/V)*1e9

# Cell death constant (units: hr^-1)
death_rate_constant = 0.2*maximum_specific_growth_rate

# Saturation constants for translation and transcription (units: nM)
saturation_transcription = 4600*(1/av_number)*(1/V)*1e9
saturation_translation = 100000*(1/av_number)*(1/V)*1e9
--------------------------------------------------------------------------------------
```

Estimation and cross-validation of EMT model parameters.

We used the Pareto Optimal Ensemble Technique (POETs) multiobjective optimization framework in combination with leave-one-out cross-validation to estimate an ensemble of TGF$-\beta$/EMT models. Cross-validation was used to calculate both training and prediction error during the parameter estimation procedure [97]. The 41 intracellular protein and mRNA data-sets used for identification were organized into 11 objective functions. These 11 objective functions were then partitioned, where each partition contained ten training objectives and one validation objective. POETs integrates standard search strategies e.g., Simulated Annealing (SA) or Pattern Search (PS) with a Pareto-rank fitness assignment [20, 96]. Denote a candidate parameter set at

iteration $i+1$ as $\mathbf{k}_{i+1}$. The squared error for $\mathbf{k}_{i+1}$ for training set j was defined as:

$$E_j(\mathbf{k}) = \sum_{i=1}^{\mathcal{T}_j} \left(\hat{\mathcal{M}}_{ij} - \hat{y}_{ij}(\mathbf{k}) \right)^2 \tag{S1}$$

The symbol $\hat{\mathcal{M}}_{ij}$ denotes scaled experimental observations (from training set j) while $\hat{y}_{ij}$ denotes the scaled simulation output (from training set j). The quantity i denotes the sampled time-index and $\mathcal{T}_j$ denotes the number of time points for experiment j. In this study, the experimental data used for model training was typically the band intensity from Western or Northern blots. Band intensity was estimated using the ImageJ software package. The scaled measurement for species x at time $i = \{t_1, t_2, .., t_n\}$ in condition j is given by:

$$\hat{\mathcal{M}}_{ij} = \frac{\mathcal{M}_{ij} - \min_i \mathcal{M}_{ij}}{\max_i \mathcal{M}_{ij} - \min_i \mathcal{M}_{ij}} \tag{S2}$$

Under this scaling, the lowest intensity band equaled zero while the highest intensity band equaled one. A similar scaling was defined for the simulation output. By doing this scaling, we trained the model on the relative change in blot intensity, over conditions or time (depending upon the experiment). Thus, when using multiple data sets (possibly from different sources) that were qualitatively similar but quantitatively different e.g., slightly different blot intensities over time or condition, we captured the underlying trends in the scaled data. JuPOETs is free or charge, open source and available for download under an MIT software license from http://www.varnerlab.org. Details of the JuPOETs implementation, including example codes are presented in Bassen et al., [96].
