# Supplementary figures and images for: Population Heterogeneity in the Epithelial to Mesenchymal Transition Is Controlled by NFAT and Phosphorylated Sp1
Source: PLoS Comput Biol. 2016 Dec 27;12(12):e1005251. doi: 10.1371/journal.pcbi.1005251 (PMC5189931; doi:10.1371/journal.pcbi.1005251)

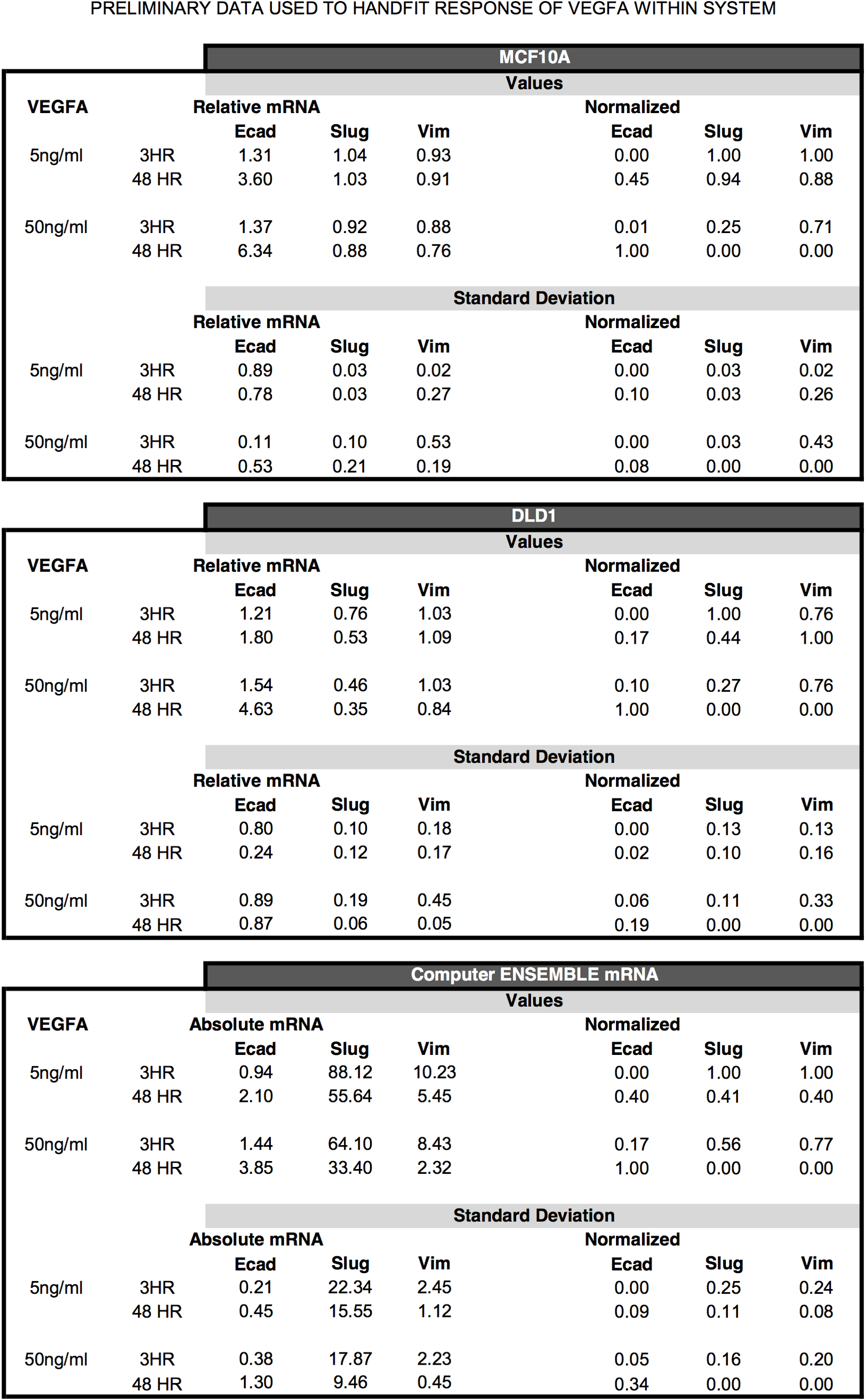

Supplement: S1 Fig — mRNA was harvested after 3hr and 48hr timepoint. (TIF) [file pcbi.1005251.s002.tif]

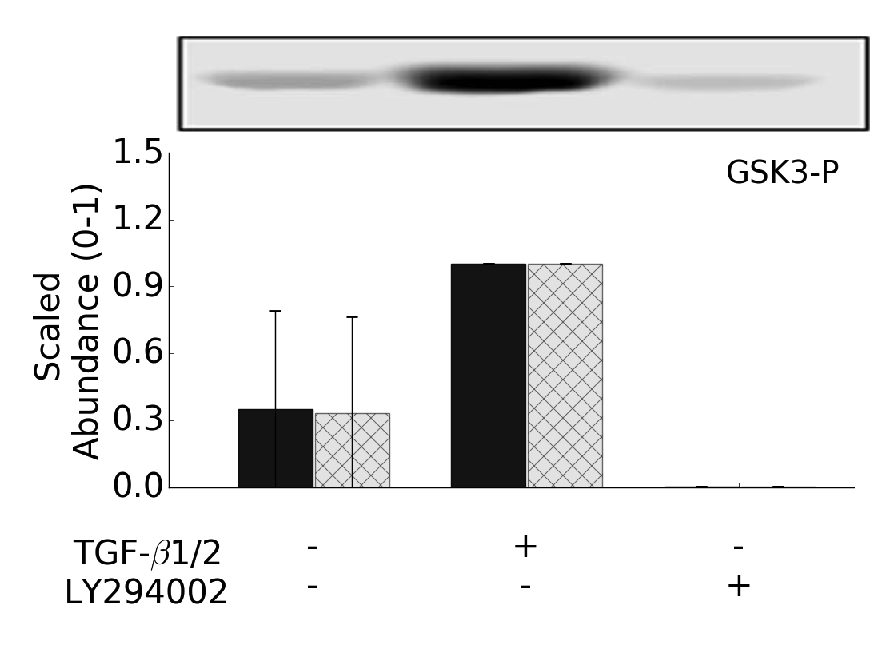

Supplement: S2 Fig — The model captured this activation through TGF−β3 signaling. LY294002 is a PI3K inhibitor. (TIF) [file pcbi.1005251.s003.tif]

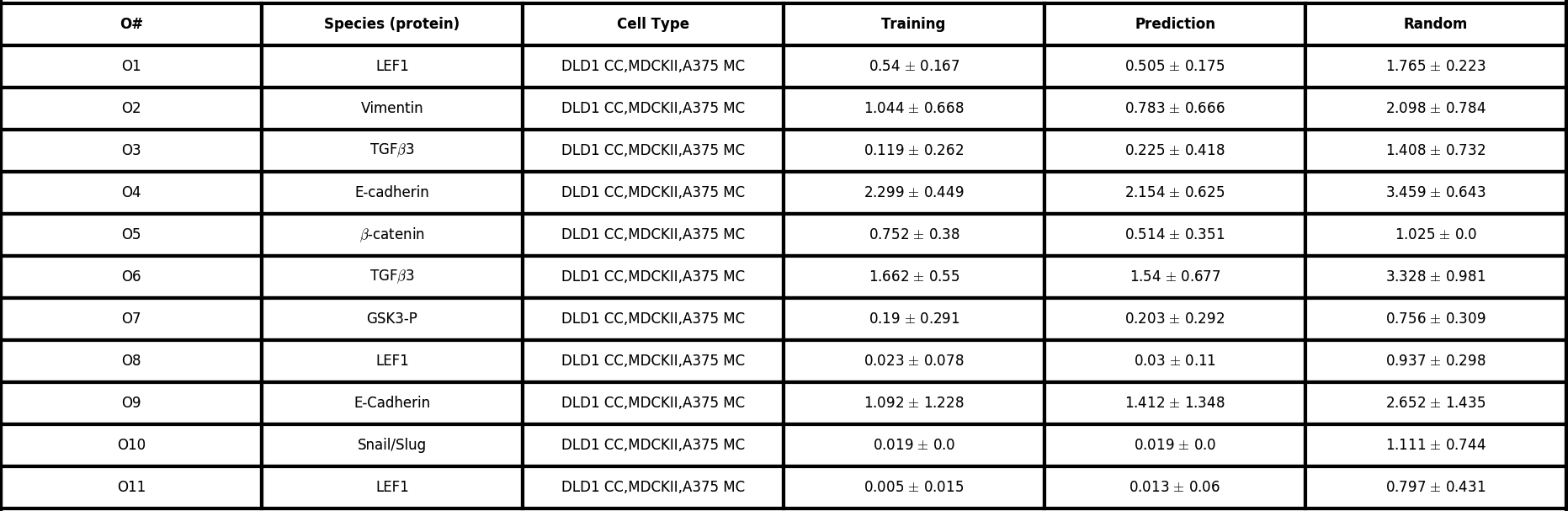

Supplement: S3 Fig — (TIF) [file pcbi.1005251.s004.tif]

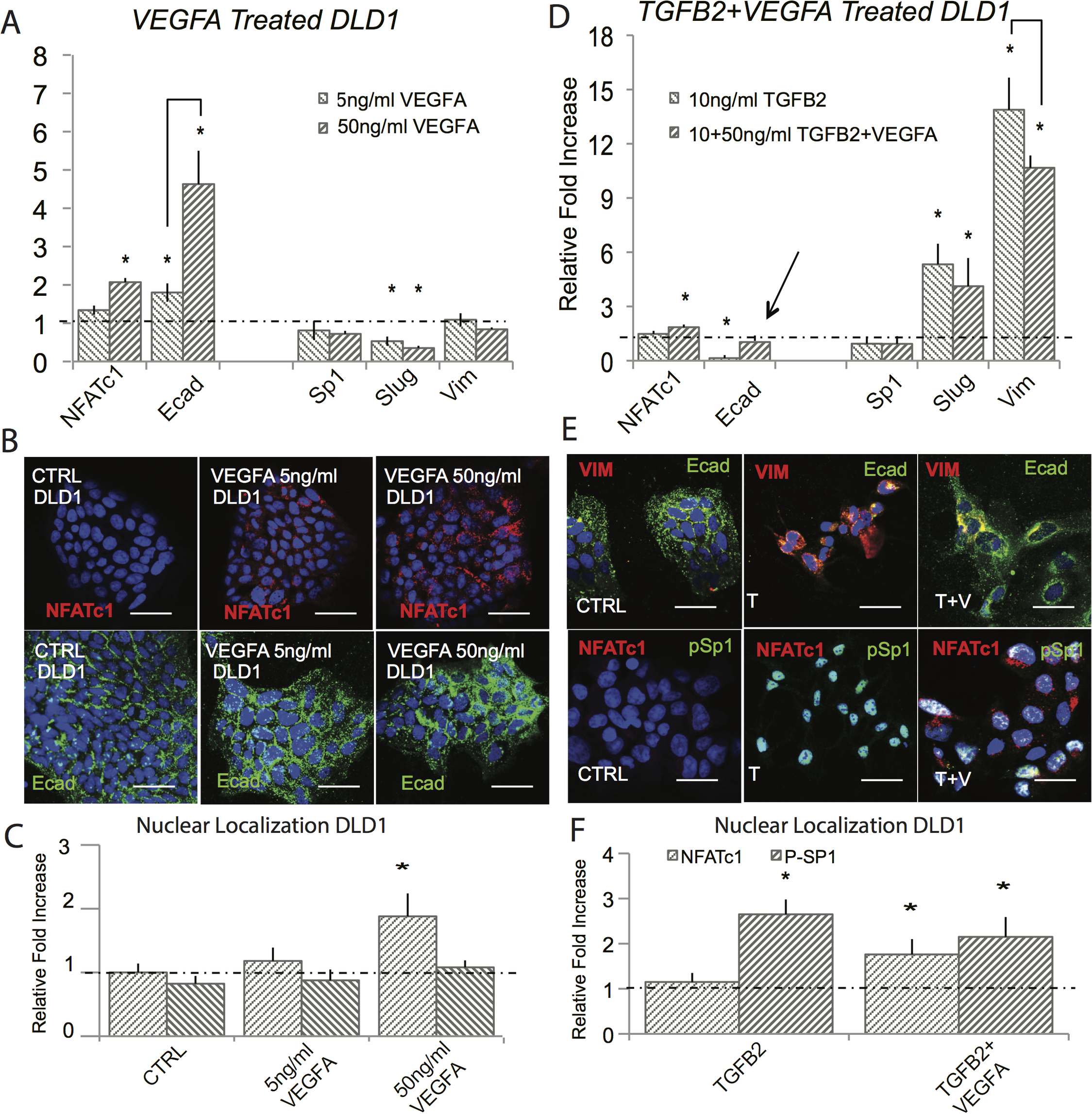

Supplement: S4 Fig — (A) In DLD1, we found that 5ng/ml of VEGFA increased NFATc1 and E-cadherin gene expression via qPCR and 50ng/ml potentiated this effect at 48 hrs. (B–C) These findings were confirmed at the protein level via immunofluorescence, as ecadherin levels and nuclear localization of NFATc1 increased. (D) Treatment with (10ng/ml) TGFβ2 resulted in mesenchymal transformation as measured via qPCR against target genes Slug, ecadherin, vimentin, Sp1, and NFATc1. (E–F) Immunofluorescence and nuclear localization revealed a strong presence of phospho-Sp1. (G) Combination of VEGFA (50ng/ml) and TGFβ2 (10ng/ml) treatment resulted in increased Slug, NFATc1, and vimentin expression, while also increasing ecadherin levels compared to control. (H) Immunofluorescence confirmed these results, as both ecadherin and vimentin levels were elevated. (I) A significant increase in nuclear localization of both NFATc1 and phospho-Sp1 were also found. Magnification, 40x. Scale bars: 50μm. C = Control, T = TGFβ2, V = VEGFA, VI = NFAT inhibitor (VIVIT). Asterisks signify statistical differences from each other according to a one-way ANOVA with Tukey’s post hoc (p≺0.05). (TIF) [file pcbi.1005251.s005.tif]

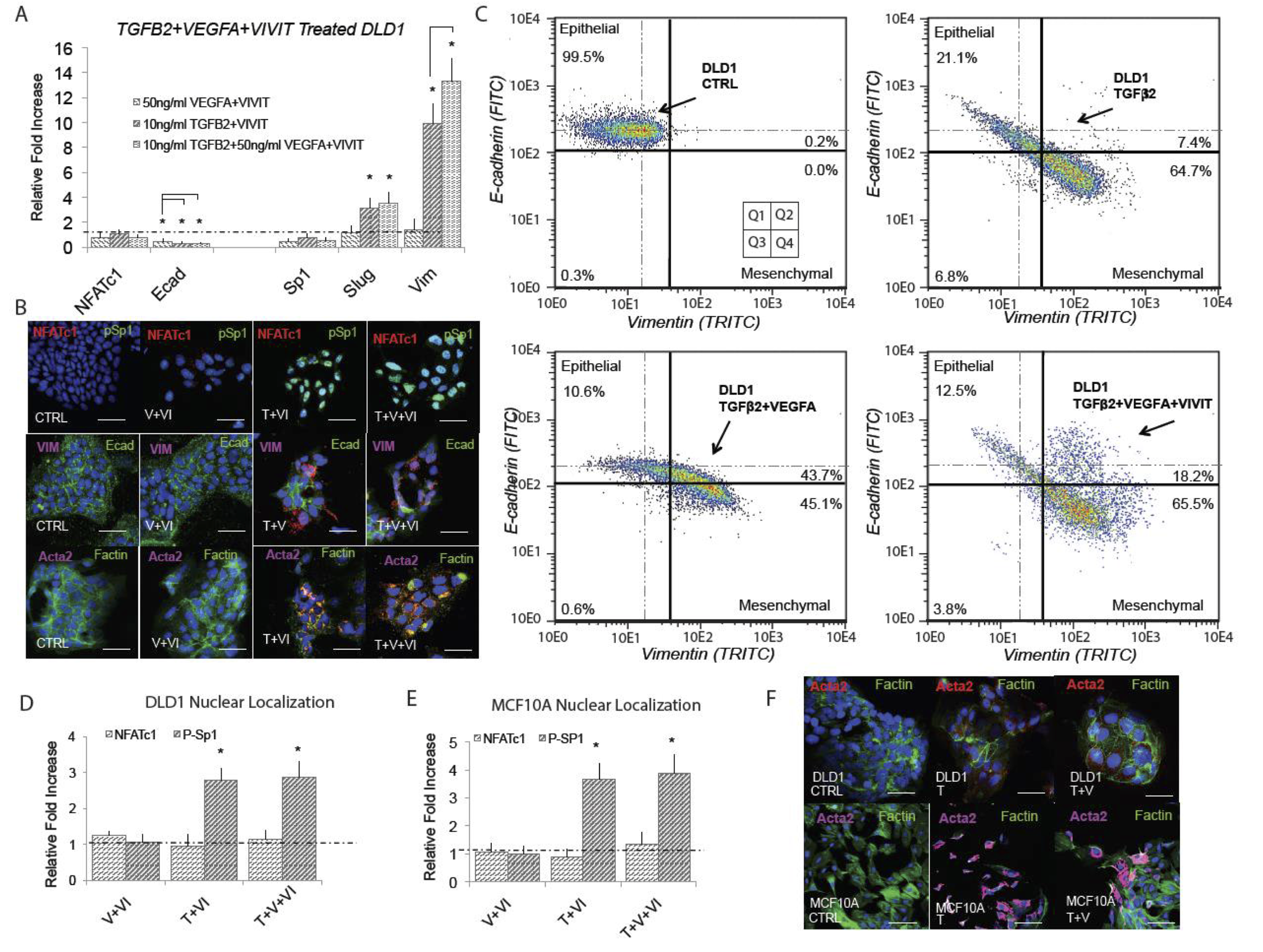

Supplement: S5 Fig — (A) Treatment with VEGFA (50ng/ml) and NFAT inhibitory peptide VIVIT (10μM) resulted in significantly reduced ecadherin expression (qRT-PCR at 48hrs). Addition of TGFβ2 (10ng/ml) and VIVIT resulted in increased Slug and vimentin expression, while inhibiting ecadherin levels. Combined TGFβ2, VEGFA, and VIVIT treatment resulted in target genes Slug and vimentin expression increased, while inhibiting ecadherin levels. No change in Sp1 or NFATc1 expression was found. (B) These findings were confirmed via immunofluorescence as the VIVIT inhibitors was shown to inhibit ecadherin levels in all three cases. We also found no change in gene or nuclear localization of NFATc1 in all three cases, while phospho-Sp1 was found to increase in both TGFβ conditions. (C) Quantitative flow cytometry also confirmed this trend. (D,E) TGFβ2, VEGFA and VIVIT treatment in DLD1 and MCF10A resulted in no change of Sp1 expression or NFATc1 expression. (F) Likewise, no change in nuclear localization of NFAT in all three cases, however phospho-Sp1 was found to increase in both TGFβ conditions. Magnification, 40x. Scale bars: 50μm. C = Control, T = TGFβ2, V = VEGFA, VI = NFAT inhibitor (VIVIT). Asterisks signify statistical differences from each other according to a one-way ANOVA with Tukey’s post hoc (p≺0.05). (TIF) [file pcbi.1005251.s006.tif]
